# COP9 Signalosome Component JAB1/CSN5 Is Necessary for T Cell Signaling through LFA-1 and HIV-1 Replication

**DOI:** 10.1371/journal.pone.0041725

**Published:** 2012-07-24

**Authors:** Shigemi M. Kinoshita, Peter O. Krutzik, Garry P. Nolan

**Affiliations:** 1 Laboratory of Immune Regulation, Osaka University Graduate School of Frontier Biosciences, Suita, Osaka, Japan; 2 Department of Pathology, Anatomy and Cell Biology, Thomas Jefferson University, Philadelphia, Pennsylvania, United States of America; 3 Department of Molecular Pharmacology, Stanford University School of Medicine, Stanford, California, United States of America; 4 Department of Microbiology and Immunology, Stanford University School of Medicine, Stanford, California, United States of America; George Mason University, United States of America

## Abstract

To determine critical host factors involved in HIV-1 replication, a dominant effector genetics approach was developed to reveal signaling pathways on which HIV-1 depends for replication. A large library of short peptide aptamers was expressed via retroviral delivery in T cells. Peptides that interfered with T cell activation-dependent processes that might support HIV-1 replication were identified. One of the selected peptides altered signaling, lead to a difference in T cell activation status, and inhibited HIV-1 replication. The target of the peptide was JAB1/CSN5, a component of the signalosome complex. JAB1 expression overcame the inhibition of HIV-1 replication in the presence of peptide and also promoted HIV-1 replication in activated primary CD4^+^ T cells. This peptide blocked physiological release of JAB1 from the accessory T cell surface protein LFA-1, downstream AP-1 dependent events, NFAT activation, and HIV-1 replication. Thus, genetic selection for intracellular aptamer inhibitors of host cell processes proximal to signals at the immunological synapse of T cells can define unique mechanisms important to HIV-1 replication.

## Introduction

In primary T cells, productive HIV-1 replication occurs only in activated T cells. Therefore essential host processes and molecules that support HIV-1 replication become uniquely available to HIV-1 during T cell activation [1], [2], [3]. This activation process is initiated by the interaction of the T cell antigen receptor (TCR) with antigen-derived peptide bound to the major histocompatibility complex (MHC) molecule on the antigen presenting cell (APC) [4]. This cell-cell interaction encourages formation of the immunological synapses that form at the interface between a T cell and an APC [5], [6]. The immunological synapse, consisting of a central cluster of TCR and an outer ring of adhesion molecules, including leukocyte function-associated antigen-1 (LFA-1), CD28, and other surface proteins, is a necessary structure for T cell activation [7], although, it is unclear how these surface molecules regulate T cell activation status. The importance of signaling events initiated at the synapse in HIV-1 replication is not well understood.

As has been previously shown, T cell activation signals allow finalization of reverse transcription, nuclear translocation, integration, and transcription from the HIV-1 promoter [2], [8], [9], [10]. Signaling systems downstream of TCR engagement, IL-2, and other surface receptors have been implicated in creating a milieu that is conducive to productive HIV-1 infection in primary T cells [11]. HIV-1 replication spontaneously occurs in many CD4^+^ T cell lines in which host molecules necessary for HIV replication are constitutively active, but does not in primary CD4^+^ T cells. Understanding such differences allows us to exploit molecular and genetic interventions to gain insight into HIV-1 biology in human cells and to provide new targets for anti-HIV therapy.

In this report, the COP9 signalosome component JAB1/CSN5 was identified as the target molecule of a peptide aptamer that inhibited HIV-1 replication in a genetic screen. JAB1 interacts with the cytoplasmic domain of the integrin LFA-1, an adhesion molecule present during formation of the immunological synapse. Engagement and activation of LFA-1 through the immunological synapse initiates relocalization of JAB1, leading to enhanced JNK activity important for early T cell activation events [12]. The selected aptamer blocked this LFA-1-induced JAB1 relocalization event and downstream JNK activity resulting in inhibition of HIV-1 replication. Therefore, the data in this report link HIV-1 replication to early T cell activation events that are concomitant with, or that follow, signaling from the immunological synapse.

## Results

### Selection of Intracellular Aptamers that Inhibit HIV-1 Transcription Through Action Upon NFAT and AP-1 Signaling Systems

**Figure 1:**
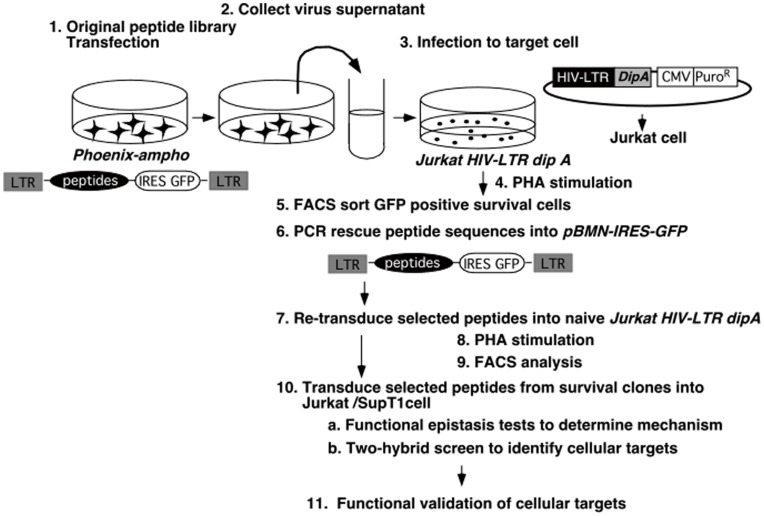
Overall strategy for screening using retrovirus peptide library. The retroviral peptide expression library is first transfected into the helper-free retroviral producer line by transient transfection. The supernatant containing the retrovirus-encoded peptide expression library is used to infect target Jurkat HIV-LTR dipA cells. The cells are subjected to PHA treatment, and GFP positive cells are selected and grown (GFP^+^ cells should be peptide expressing and should enriched after selection). Genomic DNA is derived from the sorted cells. PCR amplified DNA, using primers flanking the peptide inserts, was cleaved with restriction endonucleases and recloned into new vectors for retesting.

A dominant effector genetic screen was implemented to identify trans-acting peptides that act upon T cell signaling processes important to HIV-1 replication. The basis of the approach was retroviral expression of short peptides (10-mers) from a library of more than 10^7^ different members in T cells followed by selection for phenotypes dependent upon peptide expression. The retroviruses were designed to express both a peptide and GFP from a single transcript (Figure 1); GFP was used as a surrogate indicator of relative peptide expression in cells. The majority of peptides expressed within cells were expected to have no effect on cellular processes [13], [14], [15], and detrimental global effects on the viability of cells after expression of such libraries were not observed. As is the case with pharmaceutical screens that evaluate libraries of small organic molecules in high-throughput screening assays, certain rare peptides of the right sequence and shape were expected to interfere with intracellular signaling to give a desired phenotypic outcome when those peptides are expressed within cells.

T cell activation can be induced using the pharmacological activator PHA–this activator mimics some processes of T cell activation through action upon T cell surface receptors. The ensuing stimulation releases intracellular calcium stores and activates calcineurin and Nuclear Factor of Activated T cells (NFAT), among other events [16], [17]. The normal T cell activation program leads to activation of IL-2 and other downstream genes, but can also activate HIV-1 replication and transcription [1], [2], [9], [10], [18]. To identify peptides that dominantly interfere with T cell activation-dependent HIV-1 replication, the genetic strategy shown in Figure 1 was used. A stable Jurkat cell line was established by transfection with a dipA gene driven by the HIV-1 promoter, pHIV-LTR dipA puro^R^. This stable cell line, Jurkat HIV-LTR dipA, can be killed by stimuli that activate HIV-1 LTR activity such as PHA and Tumor Necrosis Factor-α (TNF-α) [19], [20]. These T cells were expected to survive if an expressed peptide blocked signaling that led to HIV-1 promoter activation.

A high titer retroviral supernatant of the peptide library was produced using the retroviral producer cell line Phoenix-Ampho [21] and was used to infect 3×10^8^ recipient Jurkat HIV-LTR dipA cells at about a 30% infection rate as measured by GFP expression on a per cell basis. One week after retrovirus transduction, the cells were stimulated with PHA. This stimulation was repeated six times at regular intervals over two months–each cycle of stimulation was expected to enrich for peptide-induced survivors against a background of non-heritable spontaneous survival events (i.e., cells that survived due to epigenetic events, to not receiving sufficient signal, or to other non-peptide effects). After the sixth PHA stimulation, GFP-positive surviving cells were isolated by flow cytometry. Approximately 5% of the cells were GFP-positive at this stage. After culturing the cells for an additional 14 days, total cellular DNA was prepared from these cells. Using primers specific to constant regions flanking the peptide insert, the peptide regions of the genes expressed in these cells were amplified and subcloned into pBMN-IRES-GFP retrovirus vector. Plasmid DNAs prepared from these fragments were transfected back into Phoenix-Ampho to prepare recombinant retroviral supernatants for retesting. These retroviral supernatants were transduced into fresh Jurkat HIV-LTR dipA cells as in the original screen (see above). Several clones were capable of potent inhibition of HIV replication upon HIV-1 transcription. One of these clones, Pep24, was further examined.

**Figure 2:**
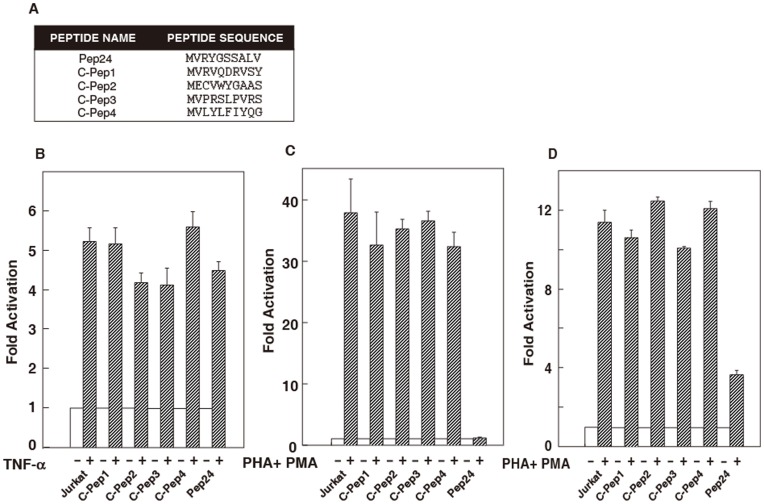
Selected peptide, Pep24, preferentially inhibits the NFAT signaling pathway. (A) The sequences of control peptides and selected peptide. (B–D) Reporter plasmids (B) p55-IgκLuc, (C) NFAT Luc, or (D) AP-1 Luc were transfected into Jurkat cells expressing indicated peptides with pBMN LacZ as the internal control plasmid. Cells treated for 3 hr (8 hr for AP-1) with or without indicated agents (2 µg/ml PHA, 10 ng/ml PMA, and 10 ng/ml TNF-α) prior to measurement of luciferase activity. The experiments were repeated three times and the average is plotted ± SE. Jurkat cells transfected with reporter without treatment were assigned a value of 1 and were used to calculate the fold activation. Transfection efficiencies were normalized to a co-transfected lacZ plasmid.

To begin to understanding how this peptide interfered with T cell activation processes, relevant signaling pathways that might be altered by peptide expression were examined. The most critical cis-regulatory elements in the HIV-1 LTR are the κB regulatory elements that can be activated by NF-κB or NFAT [9], [18], [22], [23]. The selected peptide clone, Pep24, as well as four control peptide clones (Figure 2A), were transduced into Jurkat cells using retroviral delivery, and cells were sorted by flow cytometry to select for those that expressed GFP. After culturing the selected cells and retesting, greater than 98% of cells were positive for GFP, and therefore peptide, expression. Luciferase reporter plasmids driven either by three tandemly repeated NFAT binding sites (from the IL-2 gene) [24] or by three tandemly repeated NF-κB binding sites (from the Igκ chain) [25] were transfected into these peptide-expressing cells. With TNF-α stimulation, comparable transcriptional activity was observed in clones expressing the control peptides and Pep24 and the NF-κB reporter (Figure 2B). With PHA plus PMA stimulation of cells expressing the control peptides and the NFAT specific reporter plasmid, there was the expected NFAT activation; however, no NFAT-driven transcriptional activity was observed in cells expressing Pep24 (Figure 2C). Although NFAT transcriptional activity was inhibited by Pep24, we did not observe the inhibition of NFAT nuclear translocation by Pep24 after PHA plus PMA stimulation by western blot analysis using nuclear extracts (data not shown).

We then examined the effect of Pep24 upon the AP-1 signaling pathway. AP-1 associates with the Rel domain of NFAT to control transcription of various genes upon T cell activation [16], [26], [27]. An AP-1 specific reporter plasmid (driven by five tandemly repeated AP-1 binding sites) [16] was transfected into Jurkat cells that expressed control peptides or Pep24. With PHA plus PMA stimulation, Jurkat cells that did not express peptide and the Jurkat cells expressing control peptide had similar levels of transcription activity. In Pep24 expressing Jurkat cells, however, AP-1 induced transcription activity was inhibited (Figure 2D). Thus, Pep24 inhibited AP-1 and NFAT signaling pathways but not the NF-κB pathway. Our data suggest that the inhibition of NFAT signaling by Pep24 may be due to an inhibition of AP-1 activation, as AP-1 is a fundamental part of the NFAT holoprotein complex [16], [26], [27].

### Pep24 Inhibits JNK Activity

**Figure 3:**
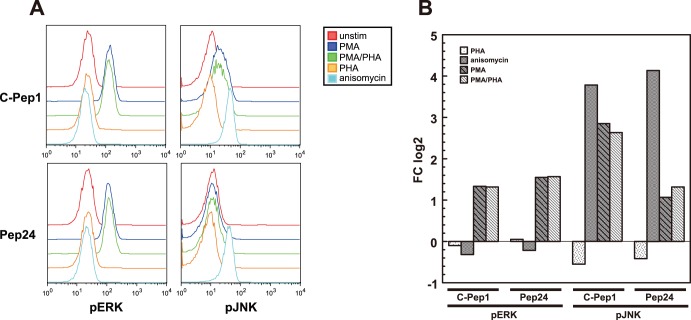
JNK is one of kinases on which HIV-1 depends. Jurkat cells expressing C-Pep1 or Pep24 were treated with indicated stimuli. Cells were stained with pERK-Alexa 647 or pJNK-Alexa 647 phosphospecific antibodies and analyzed by flow cytometry. (A) Histograms are colored according to the different stimuli. (B) Fold change was approximated by calculating the log2 ratio of mean fluorescence intensity of stimulated versus unstimulated cells.

With this initial understanding of how Pep24 interfered with T cell activation processes, the kinase activity of Jun N-terminal Kinase (JNK) and extracellular signal-regulated kinase (ERK) in Jurkat cells expressing C-Pep1 and Pep24 were examined using FACS-based intracellular phospho-protein analysis. JNK is activated by stress stimuli and pro-inflammatory cytokines and phosphorylates c-Jun and JunD, which are important components necessary for AP-1 transcription. ERK is thought to be crucial for many T cell signaling events and should serve as a control to measure how Pep24 inhibition affected other signaling events in T cells. In C-Pep1-expressing SupT1 cells, JNK was activated by PMA and PHA stimulation, but JNK activation in Pep24 expressing cells was strongly inhibited (Figure 3). Pep24 did not inhibit anisomicin-induced JNK activity. Anisomycin is an inhibitor of protein synthesis at the translation step and strongly activates stress-activated protein kinase (JNK/SAPK). Thus, these experiments indicate that JNK induction by PMA and PHA was dependent on a target molecule of Pep 24, whereas anisomicin-induced JNK activity was independent of a target molecule of Pep 24. Pep24 did not influence ERK activity (Figure 3), showing that Pep24 is not a global inhibitor of T cell signaling events.

### COP9 Signalosome Component JAB1/CSN5 as a Target of Aptamer Inhibition of HIV-1 Replication

A yeast two hybrid screen was used to identify the target molecule of Pep24. In a screening using Pep24 as bait, Jun activating binding protein (JAB1) was isolated. JAB1 was originally identified as a co-activator of the c-Jun and JunD, and was shown to be capable of acting to increase specific gene transcription driven by the AP-1 protein [28], [29]. JAB1 has also been called COP9 signalosome subunit 5 (CSN5) [30], as it is a component of the COP9 signalosome regulatory complex (CSN). The COP9 signalosome has kinase activity towards some cellular targets, including c-Jun and p53 [28], [31]. JAB1/CSN5 participates in inflammatory cascades, cell cycle regulation, apoptosis, carcinogenesis, and immunity, all as a signalosome component [12], [29], [32], [33], [34], [35].

**Figure 4:**
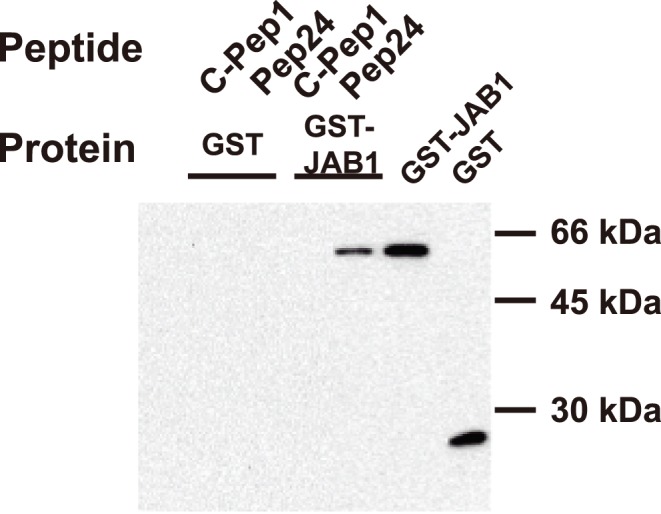
Pep24 specifically binds to JAB1. The indicated biotin-conjugated peptides were incubated with GST or with the GST-fused JAB1. The complex of peptide-GST fusion protein immobilized on streptavidin-agarose was detected by western blotting with anti-GST antibody.

To determine whether Pep24 specifically bound to JAB1, affinity binding experiments were performed using a recombinant GST-JAB1 fusion protein and biotin-conjugated peptides. Pep24 bound to the GST-JAB1 fusion protein, but did not bind to GST alone. The control peptide, C-Pep1, did not bind to either GST-JAB1 or GST (Figure 4). This indicates a specific affinity of Pep24 for JAB1 and also rules out the possibility of non-specific binding between JAB1 and the Gal4 DNA-binding domain that has caused false positive signals in the Gal-4-based yeast two-hybrid system [36].

**Figure 5:**
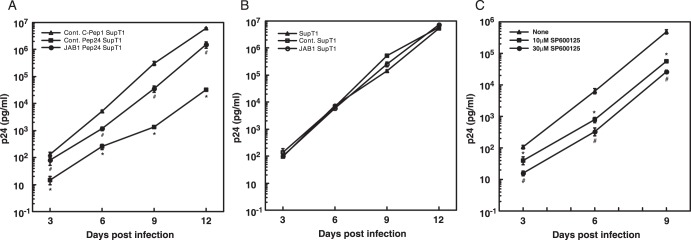
JAB1 overcomes the inhibition of HIV-1 replication in cognate peptide expressing SupT1 cells. (A) pBMN-control IRES-Lyt2α’ or pBMN-JAB1 IRES-Lyt2α’ retrovirus vectors were transduced into SupT1 cells that expressed either C-Pep1 or Pep24. (B) pBMN-control IRES-Lyt2α’ or pBMN-JAB1 IRES-Lyt2α’ retrovirus vectors were transduced into SupT1 cells. These cells were challenged with HIV-1 (NL4-3) at a dose 400 TCID_50_ per 5×10^4^ cells. p24^gag^ levels in culture supernatants were assayed from four wells on the indicated days after infection. p24^gag^ levels were normalized to cell number determined using an XTT assay. Data are presented as the average ± SE per 10^6^ cells. Similar results were observed in three independent experiments. * indicates *p*<0.05, Control C-Pep-1 SupT1 versus Control Pep24 SupT1, and # indicates *p*<0.05, Control Pep24 SupT1 versus JAB1 Pep24 SupT1 by *t* test. (C) JNK inhibitor (SP600125) inhibits HIV-1 replication. SupT1 cells were treated JNK inhibitor (SP600125) for 30 min before HIV-1 challenge. These cells were challenged with HIV-1 (NL4-3) at a dose 400 TCID_50_ per 5×10^4^ cells. p24^gag^ levels in culture supernatants were assayed from five wells on the indicated days after infection. p24^gag^ levels were normalized for cell number using XTT assay. Data are presented as the average ± SE per 10^6^ cells. Similar results were observed in three independent experiments. *indicates *p*<0.05, No treatment versus 10 µM SP600125, and # indicates *p*<0.05, No treatment versus 30 µM SP600125 by *t* test.

To examine whether Pep24 inhibits HIV-1 replication and whether inhibition can be recovered by JAB-1 expression, control (pBMN control IRES-Lyt2α’) or JAB1-expressing (pBMN JAB1 IRES-Lyt2α’) retroviral vectors were transduced with or without C-Pep1 or Pep24 expression vectors into SupT1 cells. SupT1 cells that expressed JAB1 or control constitutively (Lyt2α’ positive) and C-Pep1 or Pep24 (GFP positive) were selected by flow cytometry. These cells were then challenged by HIV-1 (NL4-3) and p24 levels were determined. Pep24 strongly inhibited HIV-1 replication when compared with cells expressing C-Pep1 (Figure 5A). JAB1 expression alone did not alter HIV-1 replication in SupT1 cells. The inhibition of HIV-1 replication by Pep24 was partially, though not completely, overcome by JAB1 expression (Figure 5A). The partial restoration of HIV-1 replication in cells expressing both JAB1 and Pep24-expressing cells is likely caused by binding of excess JAB1 to the inhibitor Pep24. Of note, JAB1 fully restored the inhibition of HIV-1 replication by another HIV-1 inhibitor peptide, Pep107, even though Pep107 did not bind JAB1 in our yeast two hybrid assay (unpublished data). Although the host target molecule of Pep107 was not identified, these data suggest that Pep107 binds to a factor in the same signaling cascade and limits the availability of functional JAB1 or that ectopic JAB1 expression can overcome a limitation imposed by Pep107 action on that unknown factor. These data support the conclusion that JAB1 is a bona fide target molecule of Pep24. Non-transduced, C-Pep1 expression or JAB1 expression alone did not alter HIV-1 replication in SupT1 cells (Figure 5B).

To examine whether JNK inhibition by Pep24 is involved in the inhibition of HIV-1 replication, we carried out an HIV-1 replication assay using JNK inhibitor, SP600125. SP600125 inhibits JNK kinase activity by competing with ATP for its binding site. SP600125 less potently inhibits activity of MAP kinases as well. SP600125 significantly reduced HIV-1 replication compared with non-treated cells at each time point in a dose-dependent manner (Figure 5C). This further supports the hypothesis that JNK, acting downstream of JAB1, is a critical kinase on which HIV-1 depends for its replication.

**Figure 6:**
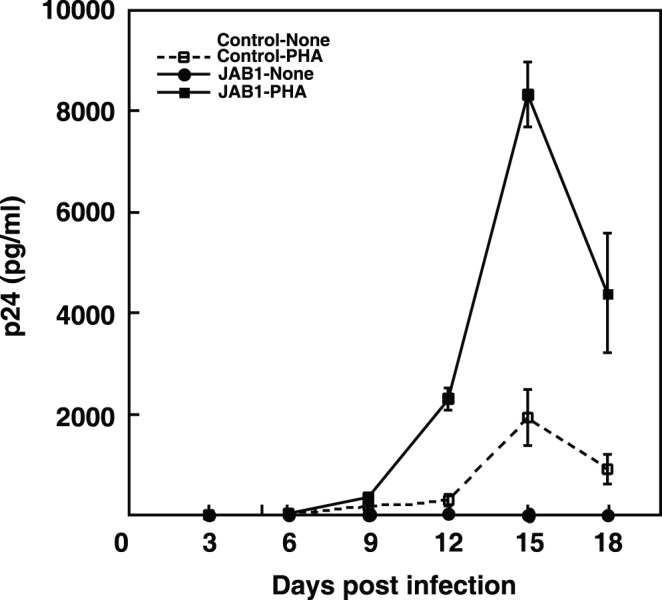
JAB1 promotes HIV-1 replication in activated primary CD4^+^ **T cells.** pBMN-control-IRES-Lyt2α’ and pBMN-JAB1-IRES-Lyt2α’ retrovirus vectors were transduced into human primary CD4^+^ T cells. CD4^+^ T cells were challenged with HIV-1 (NL4-3) at doses of 400 TCID_50_ per 1×10^5^ cells with or without PHA stimulation. p24^gag^ levels in culture supernatants were assayed from four wells on the indicated days after infection. p24^gag^ levels were normalized for cell number determined using an XTT assay. Data are presented as the average ± SE per 10^6^ cells. Similar results were observed in three independent experiments.

To determine whether JAB1 could affect HIV-1 replication in primary CD4^+^ T cells, HIV-1 replication was measured in primary CD4^+^ T cells with or without ectopic JAB1 expression. JAB1 was retrovirally transduced into primary CD4^+^ T cells. Control primary CD4^+^ T cells and JAB1-expressing primary CD4^+^ T cells were infected with NL4-3, and HIV-1 replication levels were measured by a p24 ELISA. After 18 days, most cells were dead and the experiment was terminated. No HIV-1 replication was observed in either control or JAB1-expressing non-stimulated primary CD4^+^ T cells. However, after PHA stimulation, HIV-1 replication was enhanced about four fold in JAB1-expressing primary CD4^+^ T cells in comparison with control primary CD4^+^ T cells (Figure 6). This shows that T cell activation and JAB1 activity are required for HIV-1 replication in primary CD4^+^ T cells. It can be concluded that endogenously expressed JAB1 is acted upon during T cell activation to enhance signaling important to HIV-1 replication.

### Pep24 Inhibits LFA-1-induced JAB1 Relocalization

**Figure 7:**
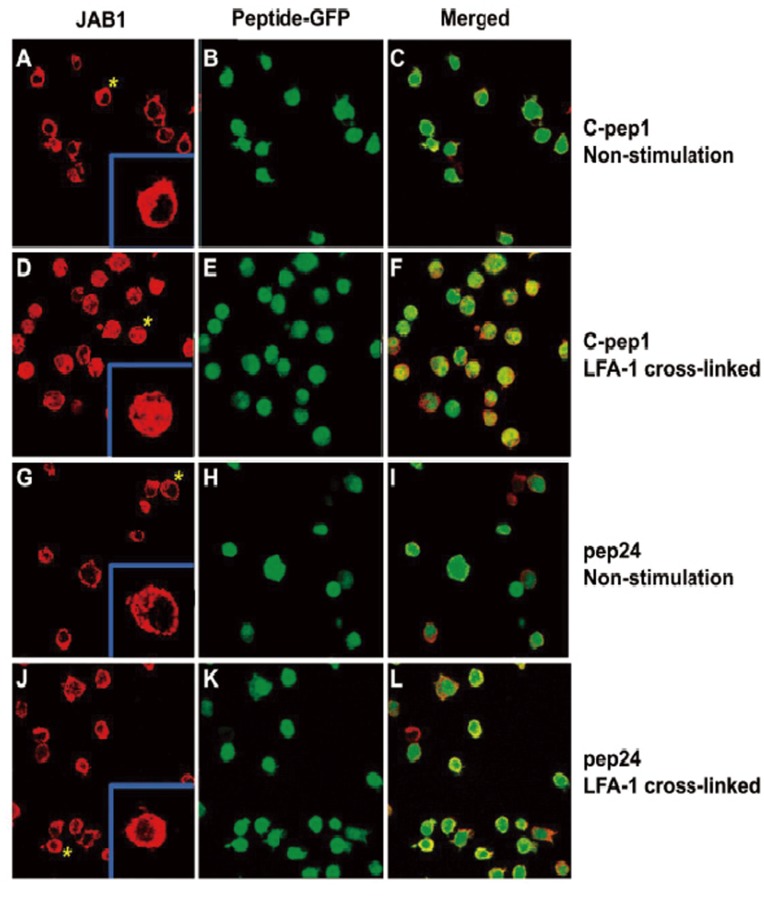
Pep24 blocks LFA-1-induced JAB1 relocalization. Jurkat cells expressing indicated peptides were adhered to either anti-LFA-1 mAb or control IgG coated plates for 30 min. After cross-linking stimulation, cells were stained for JAB1 (red). GFP (green) is an indicator of peptide expression. (A–C) C-Pep1-expressing cells without stimulation. (D–F) C-pep1-expressing cells with LFA-1 cross-linking stimulation. (G–I) Pep24-expressing cells without stimulation. (J–L) Pep24-expressing cells with LFA-1 cross-linking stimulation.

To determine how Pep24 influences JAB1 function, we examined JAB1 localization in Pep24-expressing Jurkat cells by a confocal microscope analysis. JAB1 binds to the cytoplasmic tail of the beta chain of the LFA-1 surface protein [12]. Engagement of LFA-1 with activating ligands (ICAMs 1, 2 or 3) or crosslinking with activating antibodies results in release of JAB1 into the cytoplasm and nucleus [12]. As expected, JAB1 was localized at the membrane when Jurkat cells were not stimulated (Figure 7A and G). After cross-linking with anti-LFA-1 monoclonal antibody (mAb) JAB1 was redistributed to the cytoplasm and nucleus in C-Pep1-expressing Jurkat cells (Figure 7D). In cells expressing Pep24, however, JAB1 remained at the membrane (Figure 7J), indicating that Pep24 blocks JAB1 relocalization. Taken together, this suggests that by preventing JAB1 relocalization, Pep24, leads to inhibition of AP-1 function and NFAT activity and ultimately blocks critical events required for HIV-1 replication.

## Discussion

**Figure 8:**
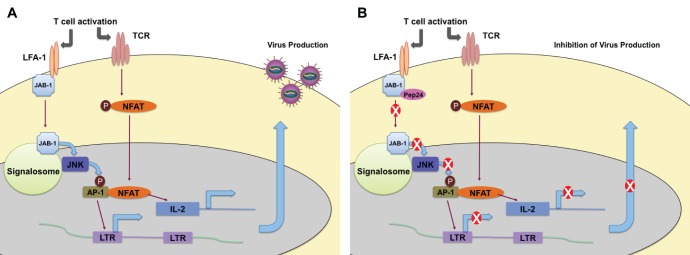
Proposed model for the inhibition of signalling pathway by Pep24. (A) After LFA-1 activation, JAB1 relocalizes into cytoplasm and nucleus, activates AP-1 and induces gene activation, and finally activates HIV-1 replication. (B) When Pep24 binds to JAB-1, JAB1 relocalization is blocked inhibiting downstream signals and HIV-1 replication.

The peptide, Pep24, was selected from a peptide library in a dominant effector genetics approach for its ability to represses host pathways that are important for HIV-1 replication in T cells. Pep24 was used as ‘bait’ to identify the host factor JAB1 as an important component that regulates HIV-1 replication in T cells. JAB1 (also known as CSN5) is involved in the activation of c-Jun, a component of the AP-1 heterodimeric transcription factor [28]. Pep24 inhibited relocalization of JAB1 post-LFA-1 engagement, downstream signaling pathways, and, therefore, HIV-1 replication (Figure 8). As JAB1 enhanced HIV-1 replication only in PHA-activated primary CD4^+^ T cells, JAB1 is a mediator of HIV-1 replication only after appropriate signaling primes its activity.

JAB1 interacts with the β subunit of the cytoplasmic domain of the integrin, LFA-1, and contributes to integrin-mediated signal transduction [12]. The interaction of LFA-1 with ICAM-1, ICAM-2, or ICAM-3 is critical for formation of the immunological synapse during full T cell activation [7], [37], [38]. The kinetics of induction of LFA-1 are complex. In resting T cells, a low affinity form of LFA-1 is present at the cell surface. The interaction of specific antigen and TCR induces intracellular signals to convert LFA-1 to a high-affinity state [37] that, in concert with engagement of ICAM ligands, can transduce signals that allow for JAB1 release. After appropriate engagement, JAB1 redistributes to the cytoplasm and nucleus where it can activate AP-1 transcriptional activity required for T cell activation [12]. Interestingly LFA-1 was previously implicated as a promoter of HIV-1 transmission during virus entry and replication [11], [39], [40], [41], [42] A mutant of LFA-1 that cannot bind with high affinity to ICAM-1 fails to promote HIV-1 replication in spite of the fact that this mutant LFA-1 promoted virus entry into cells [11], suggesting that an LFA-1 mediated signal is required to promote a replicative environment for HIV-1 in T cells. It has also been reported that HIV-1 infected, Nef-expressing macrophages shed soluble CD23 and soluble ICAM that are HIV-1 replication permissivity factors for primary resting T cells [43]. This supports our finding that the ICAM/LFA-1 interaction and the ensuing activation of the JAB1 signaling pathway are critical for HIV-1 replication in T cells. Taken together, current data suggest that the formation of the APC::T cell junction, LFA-1 engagement, and subsequent signaling that releases JAB1 and activates the transcriptional regulators AP1 and NFAT (initial immutable events during T cell activation) leads to a molecular outcome that facilitates HIV-1 replication.

JAB1 is thought to interact with many proteins such as p27^kip1^, macrophage migration inhibitory factor 1 (MIF1), c-Jun, LFA-1, Bcl-3, and steroid receptor co-activator (SRC-1) [12], [29], [32], [33], [44], [45]. The functions of JAB1 in these interactions are not well understood. JAB1/CSN5 has been shown to contain a JAMM domain and support an isopeptidase in the COP9 signalosome in the removal of the ubiquitin-like Nedd8 from the Cul1 subunit of SCF ubiquitin ligase [46]. It is known that JAB1 engagement of p27^kip1^ allows a cell to proceed through the G1 phase of the cell cycle [33]. Based on these results, we hypothesized that the binding to JAB1 by Pep24 led to a blockade of JAB1 function. If the interaction of JAB1 and Pep24 prevents the binding to p27^kip1^ by JAB1 there should be cell cycle arrest. However, this was not observed in Pep24-expressing T cell (data not shown). This suggests that Pep24 binds to a region of JAB1 that effects regulation of c-Jun but not p27^kip1^. It is also possible that the peptide binds to a region of JAB1 that must be post-translationally modified to allow its release from LFA-1. If so, the Pep24-JAB1 interaction site could be a locus for design of small molecule regulators of specific T cell functions, including those that either distinctly, or spatially, inhibit this aspect of T cell activation, to block HIV-1 replication. Importantly, JAB1 has not previously been identified as a potential drug target. As the JNK inhibitor SP600125 [47], [48], [49] blocked HIV-1 replication in a dose-dependent manner, specific MAP kinase-related events downstream of JAB1 facilitate HIV-1 replication.

In this study, intracellular peptide display technology was used to identify host factors critical to HIV-1 replication pathways. Host factors defined in this manner are candidates for drug development. Inhibitors that block virus access to necessary host factors may be less likely to result in drug resistance than drugs targeting viral proteins. This dominant effector screening approach might also be applied as an adjunct to classical analysis of signaling systems to provide a series of inhibitors that define complementation groups. A unique involvement of LFA-1 signaling via JAB1 that is involved in T cell activation and enables HIV-1 replication in cells was demonstrated. The intriguing role of JAB1 in early T cell activation events that drive HIV-1 replication underscores a need for new tools for identification of early and late host processes required for pathogen parasitism.

## Materials and Methods

### Plasmid Construction

The pHIV-LTR dipA puro^R^ consists of the HIV-1 long terminal repeat (LTR containing the enhancer and promoter region; −520/+81) linked to cDNA of the diphtheria toxin A chain gene. The polyadenylation site was derived from SV40. Downstream of this was placed the puromycin resistance gene driven from the CMV promoter and the polyadenylation site was derived from SV40. The sequence of control peptides (C-Pep1-4) and Pep24 were inserted into the retroviral expression vector pBMN-IRES-GFP using BamHI and SalI sites. The coding sequence of JAB1 was inserted into retroviral expression vector pBMN-IRES-Lyt2α’ using BamHI and SalI. pEBG-JAB1 was constructed by inserting the cording sequence of the JAB1 into a eukaryotic glutathione-S-transferase (GST) expression vector pEBG.

### Construction of Random Peptide Library

The library insert was constructed using an oligonucleotide with the following sequence 5′-AGCTAGATCGCAGTGTGCCACCATGGNK(NNK)_8_TGACTGACTGAGTCGACATCG-3′ where N was a mixture of each of the four nucleotides and K was a mixture of G and T. The DNA oligonucleotide library template was amplified by five rounds of PCR using the primers 5′-AGCTAGATCGCAGTGTGCCACCATG-3′ and 5′-CTCGAGTCAGTCAGTCA-3′. The appropriately sized fragment was gel purified and ligated into pBMN-IRES-GFP using BamHI and SalI sites. The ligated DNA was transformed into STBL4 electrocompetent *E. coli* cells (Invitrogen) according to the manufacturer’s protocol. The peptide library contained 2.9×10^7^ independent clones.

### Screening of Peptide Library and Rescue of Survivor Peptides

Recombinant retroviruses representing the peptide library were prepared using the Phoenix Ampho retrovirus packaging cell line [21] and used to infect 3×10^8^ Jurkat HIV-LTR dipA cells as described previously [9]. The cells infected with the peptide library were stimulated with 2 µg/ml phytohaemaglutinin (PHA) six times over a period of 2 months. During this selection period, cells were cultured in RPMI 1640 containing 2.5% fetal calf serum (FCS). After this selection, GFP-positive living cells were sorted by flow cytometry, and total cellular DNA was prepared from these cells using the QIAamp Blood Kit (QIAGEN). The DNA was amplified by PCR using primers described above. The PCR fragment was ligated into pBMN-IRES-GFP, and this DNA was transformed into *E. coli* as described above.

### Luciferase Assay

Jurkat cells and peptide-expressing Jurkat cells were cultured in RPMI 1640 containing 10% FCS. The luciferase assay was performed as described previously [9]. Cells (1×10^6^) were transfected with 1 µg of reporter plasmid, 1 µg of indicated plasmid, and 1 µg pBMN LacZ using the FuGENE6 transfection reagent according to the manufacturer’s protocol (Roche Applied Science). Thirty-six hours after transfection, cells were treated as indicated, and luciferase activity was measured in cell extracts. LacZ activity was assayed by standard methods.

### HIV Infection and p24 Assay

SupT1 cells (CRL-1942, ATCC) and SupT1 cells expressing peptides were cultured in RPMI 1640 medium containing 2.5% FCS. HIV-1 infection and p24 assays were performed as described previously [9], [10]. Cells were infected with HIV-1 by incubating cells with NL4-3 (for SupT1 cells, 400 TCID50/5×10^4^ cells; for primary CD4^+^ T cells, 400 TCID50/1×10^5^ cells) in 0.5 mL of culture medium at 37°C for 4 hr. After HIV-1 challenge, cells were washed with culture medium and plated in five wells of a 48-well plate (for SupT1 cells, 5×10^4^ cells/well; for primary CD4^+^ T cells, 1×10^5^ cells/well). Virus replication was measured on the indicated days after HIV-1 challenge by p24 ELISA according to the manufacturer’s protocol (ZeptoMetrix Corporation). p24^gag^ levels were normalized to cell number measured using the XTT assay.

For the replication assay in the presence of the JNK inhibitor, SupT1 cells were treated with 10 µM or 30 µM JNK inhibitor, SP600125 (S5567, SIGMA) at 37°C for 30 min before HIV-1 challenge. Treated cells were challenged with NL4-3 (400 TCID_50_/5×10^4^ cells in 0.5 mL of culture medium). Virus replication was measured on the indicated days by p24 ELISA as described above.

### Phospho-specific Flow Cytometry

Phospho-specific flow cytometry was performed as described previously [50]. Briefly, Jurkat cells expressing control (C-Pep1) or Pep24 were treated with phorbol myristate acetate (PMA) (50 nM), PHA (2 µM), the combination of PMA and PHA, or anisomycin (2 µg/ml) for 30 min. The cells were then fixed with 1.6% formaldehyde, pelleted, permeabilized with 100% methanol, and washed with staining media (phosphate buffered saline (PBS) containing 0.5% bovine serum albumin (BSA) and 0.02% sodium azide). Samples were stained with Alexa 647 labeled pERK (#612358, BD Pharmingen) or Alexa 647 labeled pJNK (#9255S, Cell Signaling) antibody (antibodies were labeled with Alexa 647 using the Invitrogen Zenon system according to the manufacture’s protocol) and analyzed on a FacsCalibur flow cytometer (BD) equipped with a 633-nm laser for detection of Alexa 647. GFP positive cells were gated, and phospho-protein levels were analyzed. Fold change (FC) was calculated as follows: FC = MFI_stim_/MFI_unstim_, where MFI is the mean fluorescence intensity. Log_2_ conversion was performed for heat map visualization.

### Yeast Two-hybrid Screening

Pep24 was fused in-frame to the Gal4 DNA binding domain through a three-glycine spacer in the pAS2-1 GAL4 plasmid [13]. The pACT2-human leukocyte library (Clontech) was used for the screening by the yeast two-hybrid method in Y190 yeast cells as described [51] with minor modifications (40 mM 3-aminotriazole was used in selection medium).

### Affinity Binding Assay

The JAB1-glutathione S-transferase (GST) fusion protein was purified using glutathione sepharose 4B (17-0756, GE Healthcare) according to the manufacture’s protocol. The GST fusion protein and biotin-conjugated peptides were incubated in binding buffer (50 mM Tris-HCl (pH 7.5), 5 mM MgCl, 100 mM NaCl, 10% glycerol, 2 mg/ml BSA, 5 mM β-mercaptoethanol) for 3 hr at 4°C with constant rotation. The complexes of GST fusion proteins and peptides were immobilized on streptavidin agarose beads (S1638, Sigma) overnight at 4°C with constant rotation. The beads were washed four times with binding buffer and were boiled in sample buffer (50 mM Tris-HCl (pH 6.8), 2% sodium dodecyl sulfate, 1% bromophenol blue, 10% glycerol, 5% β-mercaptoethanol). Eluted proteins were resolved by SDS-PAGE, and western blot analysis was performed using anti-GST antibody (G1160, Sigma) and anti-mouse-HRP conjugate (A9044, Sigma). Blots were visualized with ECL Plus Western Blotting Detection System (GE Healthcare).

### Laser Scanning Confocal Microscopy

Jurkat cells (1×10^6^) were adhered to either anti-LFA-1 mAb-coated (clone TS1/22, 20 µg/ml, Developmental Studies Hybridoma Bank) or control IgG-coated 24-well plates by mild centrifugation (200×*g*, 10 min) and incubated at 37°C for 30 min. Cells were then washed in PBS, pH 7.4 at 4°C and fixed in 1% paraformadehyde (15 min, 4°C). Cells were permeabilized for 15 min in perm buffer (0.2% saponin in PBS containing 5% FCS), washed 1X in PBS and subjected to anti-JAB1 antibody stain (SC-9021, Santa Cruz Biotechnology; 0.2 µg/50 µL perm buffer), washed 2X in perm buffer, and stained with anti-rabbit-Alexa 594 conjugate (A-11037, Molecular Probes; 0.1 µg/50 µl perm buffer). Antibody stains were performed for 30 min at 4°C in suspension. Cells were washed twice with perm buffer and adhered to 96-well glass bottom plates (BD Biosciences) coated with Prolong Gold antifade reagent (P36930, Molecular Probes) by centrifugation (450× *g*, 5 min). Cells were visualized by sequential laser excitation using a Zeiss laser scanning confocal (LSM510) and an oil immersion 63X objective. Images were acquired using Zeiss LSM510 software and compiled using Adobe Photoshop CS2.
